# *Pinctada martensii* Hydrolysate Modulates the Brain Neuropeptidome and Proteome in Diabetic (db/db) Mice via the Gut–Brain Axis

**DOI:** 10.3390/md22060249

**Published:** 2024-05-28

**Authors:** Jiayun Li, Yijun Lv, Yuanqing Wei, Xinzhi Wang, Shenghan Yan, Binyuan Zhao, Jipeng Sun, Rui Liu, Yueyang Lai

**Affiliations:** 1 Jiangsu Key Laboratory of Research and Development in Marine Bio-Resource Pharmaceutics, Nanjing University of Chinese Medicine, Nanjing 210023, China; 20223096@njucm.edu.cn (J.L.); 20210849@njucm.edu.cn (Y.L.); wei_yuanqing@njucm.edu.cn (Y.W.); 2 Jiangsu Collaborative Innovation Center of Chinese Medicinal Resources Industrialization, Jiangsu Key Laboratory for High Technology Research of TCM Formulae, Nanjing University of Chinese Medicine, Nanjing 210023, China; xinzhiwang@njucm.edu.cn; 3 School of Pharmacy, Nanjing University of Chinese Medicine, Nanjing 210023, China; 4 Zhejiang Haifu Marine Biotechnology Co., Ltd., Zhoushan 202450, China; yshwork@163.com (S.Y.); byzhao@sjtu.edu.cn (B.Z.); 5 Zhejiang Marine Development Research Institute, Zhoushan 316021, China; jipengsun@yeah.net; 6 Animal-Derived Chinese Medicine and Functional Peptides International Collaboration Joint Laboratory, Nanjing University of Chinese Medicine, Nanjing 210023, China; 7 Jiangsu Collaborative Innovation Center of Traditional Chinese Medicine Prevention and Treatment of Tumor, Nanjing University of Chinese Medicine, Nanjing 210023, China

**Keywords:** *pinctada martensii* enzymatic hydrolysates, hypoglycemic activity, gut microbiome, neuropeptidome, proteome

## Abstract

*Pinctada martensii* hydrolysate (PMH) has been proved to have the effect of ameliorating disorders of glucose and lipid metabolism in db/db mice, but the mechanism of its hyperglycemia effect is still unclear. Bacterial communities in fecal samples from a normal control group, a diabetic control group, and a PMH-treated diabetes mellitus type 2 (T2DM) group were analyzed by 16S gene sequencing. Nano LC-MS/MS was used to analyze mice neuropeptides and proteomes. The 16S rDNA sequencing results showed that PMH modulated the structure and composition of the gut microbiota and improved the structure and composition of Firmicutes and Bacteroidetes at the phylum level and *Desulfovibrionaceae* and *Erysipelatoclostridiaceae* at the family level. Furthermore, the expressions of functional proteins of the central nervous system, immune response-related protein, and proteins related to fatty acid oxidation in the brain disrupted by an abnormal diet were recovered by PMH. PMH regulates the brain neuropeptidome and proteome and further regulates blood glucose in diabetic mice through the gut–brain axis. PMH may be used as a prebiotic agent to attenuate T2DM, and target-specific microbial species may have unique therapeutic promise for metabolic diseases.

## 1. Introduction

*Pinctada martensii* (Dunker) is a species of the Pteriidae, Pterioida, Bivalvia, Mollusca, and its flesh has attracted much attention because it is rich in high-quality protein. *Pinctada martensii* has been used in China for more than 1000 years as a natural resource for both medicine and food [1]. *Pinctada martensii* flesh enzymatic peptides are enzymatic peptides obtained by *Pinctada martensii* through acid protease hydrolysis. *Pinctada martensii* flesh enzymatic peptides have been found to be beneficial for the treatment of diabetes mellitus type 2 (T2DM). T2DM is a chronic metabolic disease that accounts for more than 90% of cases of diabetes [2]. Our previous work showed that *Pinctada martensii* hydrolysate (PMH) had a hypoglycemic effect and could be used as a potential natural T2DM intervener. The hypoglycemic activity of PMH is related to its regulation of the PI3K/AKT pathway. The PI3K/AKT pathway and the retinol pathway are considered as other potential pathways for PMH to exert hypoglycemic effects [3]. However, the mechanism by which PMH exerts hypoglycemic effects is still unclear.

The gut–brain axis plays an important role in regulating various diseases. Intestinal brain interaction includes neural, endocrine, and immune pathways. The intestinal nervous system can independently regulate and control the secretion and movement of the digestive tract. The neuroendocrine pathway regulates various activities through hormones or neuropeptides. Microorganisms colonized in the intestine directly participate in the immune regulation of the body by promoting antigen secretion and regulating the production of various cytokines. The gut microbiota affects neural development, cognition, and behavior by regulating bidirectional communication between the gut and the central system. For example, a high-fat or high-carbon water diet can induce disorders in glucose and lipid metabolism, leading to disruption of the diversity and stability of the gut microbiota. Intestinal flora disorder caused by diet affects hypothalamic function by affecting vagal afferent signals, and then stimulates the occurrence of glucose and lipid metabolism disorder, leading to diseases and complications such as diabetes and hyperlipidemia.

The gut–brain axis is believed to be involved in the regulation of blood glucose. Proteins and neuropeptides in the central nervous system are involved in feeding behavior, insulin regulation, and other hormone regulation. The central nervous system, especially the hypothalamus, plays an important role in regulating appetite [4]. The gut–brain axis is a bidirectional signaling pathway regulating metabolism through balancing food intake and energy expenditure [5]. It mainly includes the brain, brain-derived neurotrophic factors, gut microbes, the gut and its secreted neuropeptides, and the autonomic nervous system [6]. The gut–brain axis is intimately involved in regulating glucose homeostasis, and this system plays a key role in mediating the efficacy of therapeutics that have had a major impact on treating T2DM [7].

The gut microbiota is considered a vital influence on T2DM; a previous study found that the development of T2DM and the mechanism of its therapeutic drugs may be related to the gut microbiome [8]. The pathogenesis of insulin resistance and type 2 diabetes mellitus (T2DM) has always been the focus of researchers. Recent research suggests that the gut microbiota may play a crucial role in this process. The gut microbiota can produce various substances, such as peptidoglycans and lipopolysaccharides (LPS), which can cause peripheral tissue inflammation and enter the bloodstream, leading to insulin resistance and T2DM. Therefore, from a perspective focusing on the gut microbiota, studying its impact on the mechanism of PMH becomes a potential approach to understanding insulin resistance and T2DM. Furthermore, this also reveals the importance of microbial communities in health maintenance. Accumulated evidence has suggested that the gut microbiota can affect the host’s physiological status through gut–brain axis pathways [9,10]. *B. longum* CKD1 enhances the efficacy of anti-diabetic medicines through upregulation of the IL- 22 response in type 2 diabetic mice [11]. *Bacteroides Ovatus* is a beneficial bacterium that can improve blood lipids and reduce weight [12]. 

Previous studies have shown that gut microbes can regulate central proteins and neuropeptides through the gut–brain axis, and the regulation of proteins may involve pathways related to hypoglycemia, or neuropeptides are closely related to animal feeding behavior, mood, lipid metabolism, hormone metabolism, insulin regulation, etc. Therefore, we speculate that regulating gut microbiota can regulate the central nervous system, and changes in central proteins and neuropeptides can participate in the regulation of blood sugar. The disruption of the gut–brain axis may be a potential cause of T2DM, but the specific signaling mechanisms in the gut–brain axis are still unclear. Therefore, it is necessary to find an effective and relatively safe treatment or improvement method for T2DM, as well as to gain a deeper understanding of the underlying mechanisms of the bidirectional connection in the gut–brain axis.

Here, 16S ribosomal DNA (rDNA)-based microbiota analysis was used to analyze the composition of intestinal microbiota in mice feces. In addition, nano LC-MS/MS was used to analyze and identify the neuropeptides and proteins in mice brain tissue. This work aims to explore the mechanism of PMH in improving glucose metabolism in db/db mice through the gut–brain axis. These findings may provide stronger support for the development of peptides derived from marine flesh as the theoretical basis for hypoglycemic products.

## 2. Results and Discussion

### 2.1. Identification and Characterization of PMH

A total of 263 peptides were identified, mainly from histone, microtubules, and actin, in PMH. The ratio of hydrophobic to hydrophilic amino acids in these peptides is about 1.07:1. A total of 55.89% of the peptides have hydrophilicity, and the molecular weight of the peptides is mainly distributed between 796.44 and 2867.34 Da. A total of 85.17% of the peptides have a molecular weight below 2000 Da. These results indicate that PMH is highly stable in the gastrointestinal tract and has potential as an oral dietary supplement. Our previous studies have shown that PMH can significantly reduce the blood glucose levels of db/db mice and improve insulin resistance and lipid metabolism in db/db mice after four weeks of intragastric administration, which works through PI3K/Akt signaling. We also found that the retinol pathway is another potential pathway for PMH to exert hypoglycemic effects through LFQ proteomics of mouse liver tissue [3]. However, the mechanism of its hypoglycemic effects is still unclear, and we are trying to reveal its possible mechanism of action through the gut–brain axis: PMH acts on the gut microbiota, then travels up through the gut–brain axis and exerts hypoglycemic effects by affecting the release of central hormones or neuropeptides.

### 2.2. Gut Microbial Diversity Analysis

**Figure 1:**
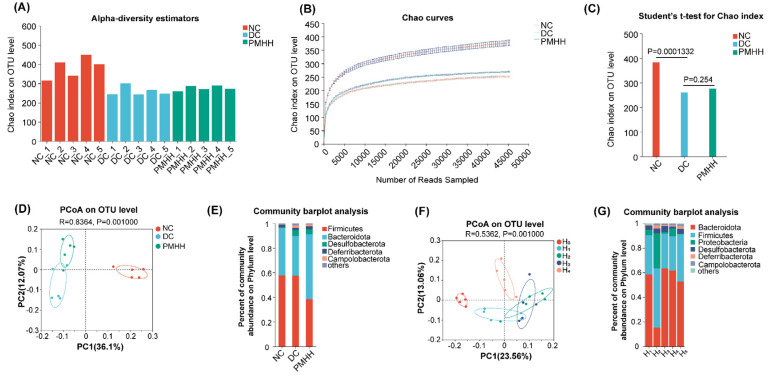
Diversity of fecal microbiota among the NC, DC, and PMHH groups. Chao index (data expressed as mean ± SD) (**A**), estimator stat (**B**), rarefaction analyses (**C**), multivariate analysis of variance from unweighted UniFrac-based PCoA matrix scores (**D**), between NC, DC, and PMHH groups, bacterial taxonomic profiling in the phylum level of the gut microbiota at the individual level (**E**), between NC, DC, and PMHH groups, multivariate analysis of variance from unweighted UniFrac-based PCoA matrix scores (**F**), between H_0_ and H_4_ groups, bacterial taxonomic profiling in the phylum level of the gut microbiota at the individual level (**G**), between H_0_ and H_4_ groups.

Common microbial α-diversity indexes, including the Chao index, rarefaction curve, and estimator stat, were evaluated (Figure 1A–C). As shown in Figure 1A, four weeks after administration, the Chao indexes of the DC (high-fat diet and pure water) and PMHH (high-fat diet and PMH, 900 mg/kg per day for 4 weeks) groups were significantly lower than those of the NC group (db/m mice serving as normal controls). Moreover, the quantity of observed OTUs in the db/db group was lower than in the other two groups (Figure 1C). In conclusion, DC displayed a decreasing biodiversity when compared with NC, and PMHH can improve the biodiversity in T2DM mice.

**Figure 2:**
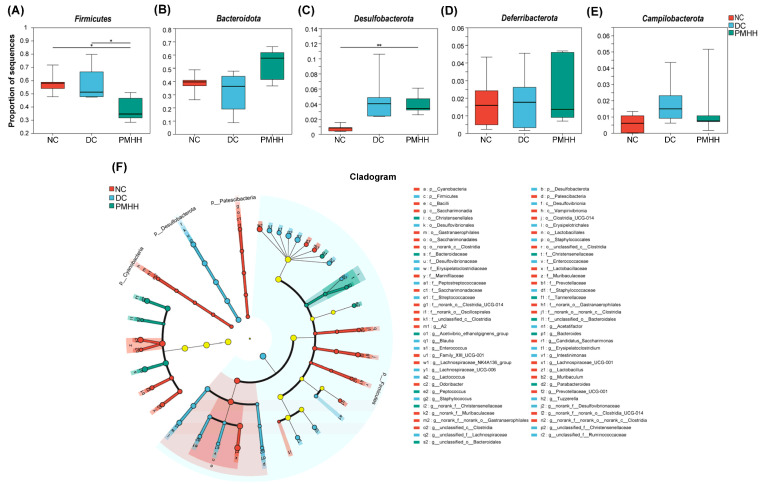
Taxonomic differences in fecal microbiota between the NC, DC, and PMHH groups and comparison of the fecal microbial communities among the three groups. Expression of Firmicutes (**A**), Bacteroidota (**B**), Desulfobacterota (**C**), Deferribacterota (**D**), and Campilobacterota (**E**) in NC, DC, and PMHH groups; taxonomic cladogram obtained by LEfSe. Differences are represented by the color of the most abundant class. (Red indicates NC, blue DC, and green PMHH.) The diameter of each circle is proportional to the taxon’s abundance (**F**). Differences were assessed by ANOVA followed by Tukey’s post hoc test and are denoted as follows: * *p* < 0.05, ** *p* < 0.01 (*n* = 5).

A UniFrac-based PCoA of the microbiota composition showed a noticeable clustering for each group (Figure 1D). The PMHH group of mice also showed significant changes in bacterial clustering within 4 weeks of administration (Figure 1F). Bacterial sequences from the three groups were analyzed at the phylum level, and the top six phyla in the relative abundance of gut microbiota in different groups and each sample are shown as follows (Figure 1E). The relative abundances of five primary phyla (*Bacteroidota*, *Firmicutes*, *Desulfobacterota*, *Deferribacterota*, and *Campilobacterota*) in each group are listed in Table S1. The relative abundance of *Bacteroidota* and *Deferribacterota* significantly decreased in DC compared with NC, and both phyla of microbiota increased after treatment with PMHH compared to DC (Figure 2B,D). The trend of changes in *Bacteroidota*, *Desulfobacterota*, and *Campilobacterota* is exactly the opposite (Figure 2A,C,E). In addition, the proportion of *Bacteroidetes*/*Firmicutes* in the feces of mice in the PMHH group also decreased compared to those in the DC group at the fourth week of administration, and at the fourth week of administration (H_4_), the proportion of *Bacteroidetes*/*Firmicutes* in the feces was decreased compared to that at the tenth week of administration (H_0_) (Figure 1G). Previous data demonstrated that *Bacteroidota* are SCFA producers [13], which have been reported to exert beneficial effects on the intestinal barrier and reduce metabolic dysfunction. Taxonomic profiling demonstrated that PMHH treatment could modulate the gut composition of mice with T2DM into a similar level to NC.

To further investigate differences in the microbiome among the NC, DC, and PMHH groups, we used LEfSe to identify the specific altered bacterial phenotypes (from phylum to family). The cladogram showed the dominant bacteria in each group (Figure 2F). Constitutions of gut bacterial species among the three groups showed apparent variation. At the general level, differential microbial lineages in the NC group included *Oscillospirales*, *Clostridia*, *Bacilli*, *Lactobacillales*, *Lactobacillacese*, *Patescibacteria*, *Saccharimonadia*, *Saccharimonadales*, *Saccharimonadaceae*, *Cyanobacteria*, *Vampirivibrionia*, *Gastranaerophilales*, *Prevotellaceae*, *Marinifilaceae*, and *Muribaculaceae*. Clades associated with DC included *Firmicutes*, *Erysipelotrichales*, *Erysipelatoclostridiaceae*, *Enterococcaceae*, *Streptococcaceae*, *Staphylococcales*, *Staphylococcaceae*, *Desulfobacterota*, *Desulfovibrionia*, *Desulfovibrionales*, and *Desulfovibrionaceae*. Moreover, *Christensenellaceae* was the most abundant in the PMHH group. It was speculated that *Christensenellaceae* might reduce inflammatory damage to the retina through the “intestinal–retinal axis”, thereby affecting disease progression in diabetic retinopathy (DR) [14]. This conjecture is consistent with the previous research results of our research group that PMHH ameliorates T2DM by regulating retinol metabolism pathways.

The changes in the microbiota after PMHH treatment were also exhibited at different taxon levels, including phylum and family. In summary, compared with NC, DC showed decreased levels of *Muribaculaceae*, *Marinifilaceae*, *Clostridia*, and *Saccharimonadaceae* (*Patescibacteria*) (Figure 2C,D), and *Desulfovibrionaceae*, *Erysipelatoclostridiaceae*, *Streptococcaceae*, and *Peptoatreptococcaceae* (*Firmicutes* and *Desulfobacterota*) were enriched in DC (Figure 2C,D). *Muribaculaceae* belongs to the phylum *Bacteroidetes*, which are beneficial bacteria and can produce SCFAs. *Muribaculaceae* are reportedly widespread in the intestinal flora and are functionally diverse in degrading carbohydrates [15]. Recent studies have demonstrated that higher abundance of *Muribaculaceae* positively correlates with the activation of the PI3K/Akt signaling pathway [16]. Bile acids (BAs) can control metabolic pathways by acting as signaling molecules that regulate triglyceride, cholesterol, glucose, and energy homeostasis [17]. *Clostridia* is one of the microorganisms that plays a major role in the BA pathway [18]. Primary BAs bind to the nuclear hormone farnesoid X receptor (FXR) [19], leading to the production of fibroblast growth factor 19 (FGF19). FGF19 can enter the systemic circulation, cross the blood–brain barrier (BBB), and activate the arcuate nucleus of the hypothalamus. This hypothalamic activation improves regulation of glucose metabolism and suppression of HPA activity. The Takeda G protein-coupled receptor 5 (TGR5) in the ileum is activated by secondary bile acids that only grow in intestinal bacteria, increasing the release of GLP-1 by L cells to regulate ingestive behavior and food intake, playing a role in controlling glucose homeostasis [20]. These changes in the microbiome indicate the gut microbiota dysbiosis in T2DM, but PMHH can ameliorate the disorder.

### 2.3. Label-Free Quantification of Endogenous Peptides in the Hypothalamus

In total, 1045 endogenous peptides derived from 32 precursors from the brain regions of NC, DC, and PMHH mice were identified. A complete summary of all the identified peptides is provided in Table S2. Highly confident peptide identification was achieved by high-resolution accurate-mass MS/MS analysis using a nano-LC Orbitrap platform.

Among the 1045 identified endogenous peptides, 1006 peptides were detected in the NC mice samples and were mainly processing products derived from well-known neuropeptide precursors, including Secretogranin-1 (SCG1), Cholecystokinin (CCKN), Thymosin beta-10 (TYB10), Neurotensin/neuromedin (NEUT), pituitary adenylate cyclase-activating polypeptide (PACA), ProSAAS (PCS1N), Pro-neuropeptide (NEUY), Secretogranin-2 (SCG2), and Prepronociceptin.

A total of 1002 peptides were detected in the db/db mice samples, including mainly SCG1, CCKN, Thymosin beta-4 (TYB4), neuroendocrine (NEC2), Proenkephalin-A (PENK), and Vasopressin–neurophysin (MEU2). A total of 1003 peptides were detected in the PMHH mice samples and were mainly processing products derived from well-known neuropeptide precursors, including SCG1, SCG2, TYB4, NEC2, NEUY, and PCS1N. A total of 395 peptides were identified in mouse samples from the NC, DC, and PMHH groups, where the PCS1N precursor accounted for the highest number of peptides (66), followed by SCG1 (62), SCG2 (61), TYB4 (31), NEUY (30), and TYB10 (23).

**Figure 3:**
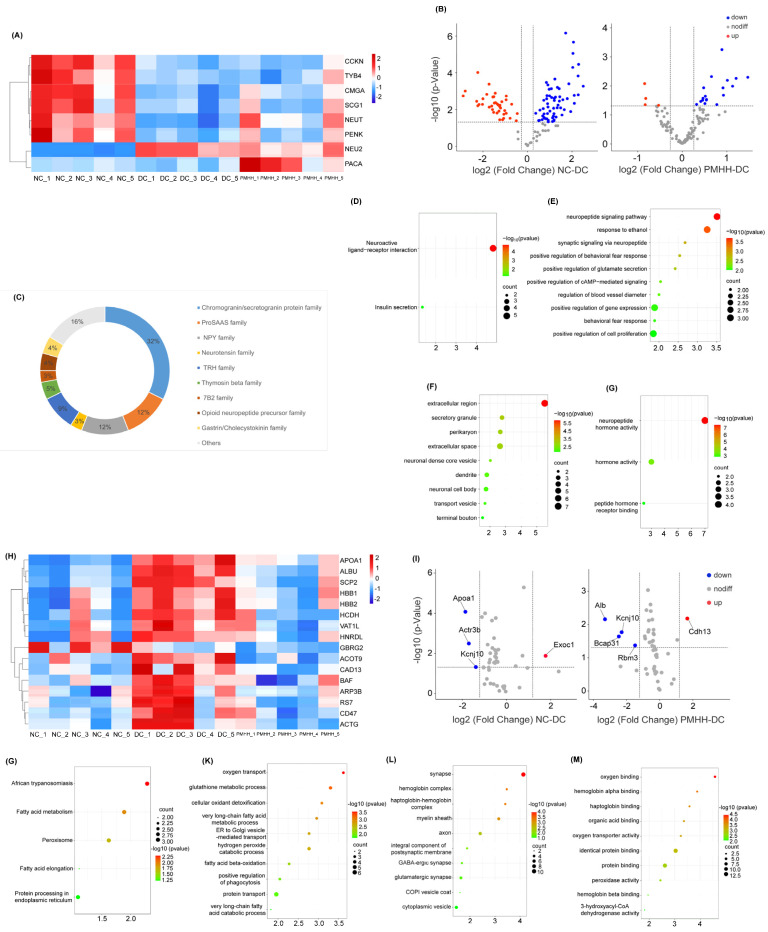
Screening of differential peptides. Heat map of differential peptide precursor proteins (**A**); differential peptide volcano plot between NC group and DC group, and DC group and PMHH group (**B**); families to which the neuropeptides belong (**C**); distribution of KEGG pathways of all the differentially expressed proteins, obtained using Omicshare online search tool (**D**); distribution of BP (**E**), CC (**F**), and MF (**G**) of all the differentially expressed proteins, obtained using Omicshare online search tool; screening of differential proteins and enrichment analysis. Heat map of differential proteins (**H**); differential protein volcano plot between NC group and DC group, and DC group and PMHH group (**I**); distribution of KEGG pathways of all the differentially expressed proteins, obtained using Omicshare online search tool (**J**); distribution of BP (**K**), CC (**L**), and MF (**M**) of all the differentially expressed proteins, obtained using Omicshare online search tool.

The LFQ of endogenous peptides was carried out in this study to compare the levels of peptides in the NC, DC, and PMHH mice samples. Peptides measured with ≥1.5-fold changes and Student’s *t*-test *p*-values < 0.05 were determined. Compared with the NC group, the intensity of 51 peptides was significantly upregulated and 15 peptides were significantly downregulated in DC mice samples. After administration of PMHH, the strengths of these peptides were significantly reversed (*p* < 0.05). These significantly changed peptides were mainly from NEUT, Chromogranin-A(CMGA), TYB4, NEU2, CCKN, PENK, PACA, and SCG1 (Table S3). Figure 3A shows the heat map of differential peptide precursor proteins. Peptides derived from SCG1, PCSK1N, and PENK were generally present at higher levels in DC mice, whereas peptides derived from CMGA, SCG2, and NEUY were usually higher in NC mice. Volcano plotting was used to compare the fold changes (Figure 3B) and the statistical significance (−log10 of the *p*-value) for these quantified proteins. We also summarized the families represented of these peptides (Figure 3C). Central administration of CCKN suppresses food intake, and there is a synergistic interaction between leptin and CCKN, irrespective of whether the leptin is delivered peripherally or into the brain [21]. NEUT not only has the function of regulating major homeostasis (heat production, pain, sleep control, and blood pressure support), but also has a positive effect in controlling food intake and energy balance [22,23,24]. Studies have shown that NEUT plays an important role in insulin resistance-related diseases such as obesity, diabetes, and non-alcoholic fatty liver disease [25]. There is evidence to prove that the arcuate nucleus (ARC) mRNA level of Proenkephalin-A is directly related to the amount of food consumed and/or changes in body weight in food-restricted and food-deprived rats [26]. The effects of PACA on food intake and body weight have been shown to depend on glutamatergic signal transduction and activation of ARC neurons to regulate food intake [27]. The above results indicate that PMH may exert hypoglycemic effects through neuropeptides involved in food intake, insulin regulation, and regulation of glucose and lipid metabolism.

KEGG analysis of the precursor proteins of these neuropeptides revealed a total of 124 pathways (*p* < 0.05, Figure 3D). Two of these pathways are neuroactive ligand receptor interaction and insulin secretion, respectively. Central action insulin acts on neurons and glial receptors widely expressed in the hypothalamus, cerebellum, cerebral cortex, and subcortical regions, thereby regulating physiological and behavioral changes. The state of insulin resistance in the brain may lead to the interruption of the insulin signaling pathway, thereby affecting a series of related dietary behaviors, energy balance, and peripheral metabolism [28]. GO enrichment analysis obtained a total of 31 biological processes or pathways, of which 17 were related to biological processes (BP) (Figure 3E), 11 were related to cellular components (CC, Figure 3F), and 3 were related to molecular function (MF, Figure 3G). BP mainly involves biological processes such as the neuropeptide signaling pathway, syntactic signaling via neuropeptides, and positive regulation of glucose secretion. CC mainly involves cellular components such as the extracellular region, secretory granule, and perikaryon. MF mainly involves functions such as neuropeptide hormone activity, hormone activity, and peptide hormone receptor binding.

### 2.4. Proteomic Analysis

Proteomic analysis of brain tissue showed that 3529 proteins were identified from the NC group, 2184 proteins were identified from the DC group, and 2377 proteins were identified from the PMHH group. The identification results for hypothalamic proteins in each group are shown in Table S4. Figure 3H shows the heat map of differential proteins. Volcano plotting was used to compare the fold changes (Figure 3I) and the statistical significance (−log10 of the *p*-value) for these quantified proteins. A *p*-value of < 0.05 and a 1.2-fold change were set as thresholds for statistically significant identifications. The 17 up- or downregulated proteins passing these thresholds are listed in Table S5. The function of aromatase (CP19A) is to catalyze the formation of aromatic C18 estrogens from C19 androgens. T2DM alters the expression of aromatase in both the central and peripheral nervous systems, and these effects of diabetes differ with gender, which was confirmed by Nihan Burul-Bozkurt. Leukocyte surface antigen CD47 (CD47) is an integral glycoprotein cell receptor and classified in the immunoglobulin superfamily [29], which is upregulated in conditions of aging and obesity. Absence of CD47 maintains brown fat thermogenic capacity and protects mice from aging-related obesity and metabolic disorder [30]. Short-chain 3-hydroxyacyl-CoA dehydrogenase (HCDH) is the enzyme involved in fatty acid oxidation. The level of HCDH was negatively associated with insulin sensitivity and positively correlated with 2-h plasma glucose levels [31].

### 2.5. KEGG Pathway Enrichment Analysis

KEGG pathway enrichment analysis of brain tissue showed that five KEGG-enriched signaling pathways were obtained (*p* < 0.05, Figure 3J). The results indicated that 17 differential proteins were mostly involved in fatty acid metabolism, peroxisome function, fatty acid elongation, and protein processing in the endoplasmic reticulum. Disturbances in fatty acid and cholesterol metabolism are evident in obesity and likely intricately linked to the development and/or sustainment of metabolic inflammation and insulin resistance [32,33]. Peroxisome proliferator-activated receptors (PPARs) regulate the gene expression involved in lipid metabolism, inflammation, and adipogenesis. Activation of PPARγ has become a target of interest to counter hyperglycemia linked with metabolic syndrome (MetS) and T2DM [34]. Increased PPARγ-driven glucose transporter-4 (GLUT4) expression enhances insulin-dependent glucose uptake [35]. Chronic endoplasmic reticulum (ER) stress and sustained activation of unfolded protein response (UPR) signaling contribute to the development of T2DM in obesity. ER stress during obesity can be beneficially rewired to promote glucose homeostasis [36]. The results of KEGG enrichment analysis of brain tissue showed that PMH mainly exerted its hypoglycemic effects by participating in fatty acid metabolism, peroxisome function, and fatty acid elongation.

### 2.6. GO Enrichment Analysis

**Figure 4:**
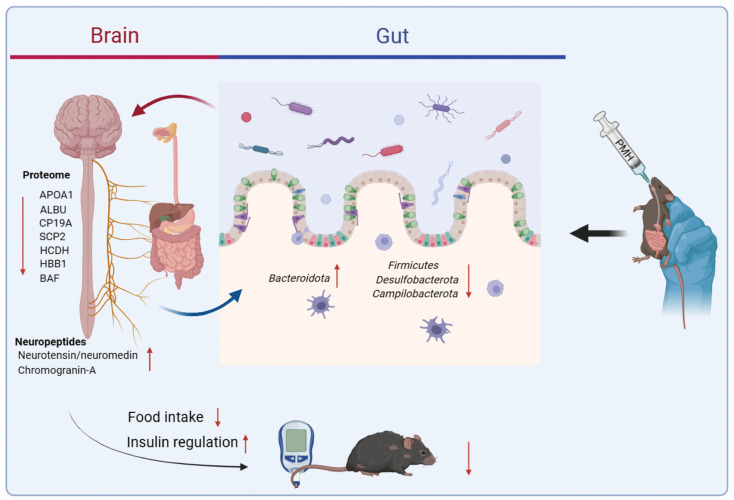
The potential mechanism of PMH exerting hypoglycemic effects through the “gut–brain axis”.

A total of 41 GO enrichment entries were obtained (*p* < 0.05), including 21 BP-related items, involving oxygen transport, the glutathione metabolic process, cellular oxidant detoxification, the very long-chain fatty acid metabolic process, and ER to Golgi vesicle-mediated transport (Figure 3K). There were 10 items related to CC, including the synapse, hemoglobin complex, haptoglobin–hemoglobin complex, myelin sheath, axon, and so on (Figure 3L). There were 10 items related to MF, including oxygen binding, hemoglobin alpha binding, haptoglobin binding, organic acid binding, oxygen transporter activity, and others (Figure 3M). Glutathione (GSH), often referred to as “the master antioxidant”, participates not only in antioxidant defense systems, but many metabolic processes. Decreased levels of GSH are likewise associated with diabetes [37]. The thiols cysteine and GSH have been reported to protect rats against the development of alloxan diabetes when injected together with alloxan [38]. It is reported that inactivation of glutathione synthesis and thiol transport in diabetic patients increases the sensitivity of the cells to oxidative stresses, and these changes may lead to the development of some complications in diabetes mellitus [39]. The potential mechanism by which PMH exerts its hypoglycemic effect through the gut–brain axis is shown in Figure 4.

## 3. Materials and Methods

### 3.1. Materials and Sample Preparation

*P. martensii* flesh was collected from Beihai, Guangxi Province (China), and kindly characterized by Professor Hao Wu, the Director of Jiangsu Key Laboratory of Research and Development in Marine Bio-resource Pharmaceutics. The *P. martensii* flesh was frozen and stored at −80 °C in the laboratory, School of Pharmacy, Nanjing University of Chinese Medicine. PMH was made in the laboratory: In order to remove polysaccharides and other water-soluble components, *P. martensii* flesh was cut into small pieces and decocted twice, one hour each time. In addition, an appropriate amount of *P. martensii* meat was homogenized by adding 3.5 times the amount of water. The pH of the homogenate was adjusted to 3.0, and the acid protease was added at a dose of 1000 U/g (Beijing Solarbio Science & Technology Co., Ltd., Beijing, China). After enzymatic digestion at 45 °C for 3 h, the enzyme was inactivated in a boiling water bath for 10 min. The supernatant was centrifuged and then lyophilized and kept at −20 °C until use.

### 3.2. Animal and Experimental Procedures

**Figure 5:**
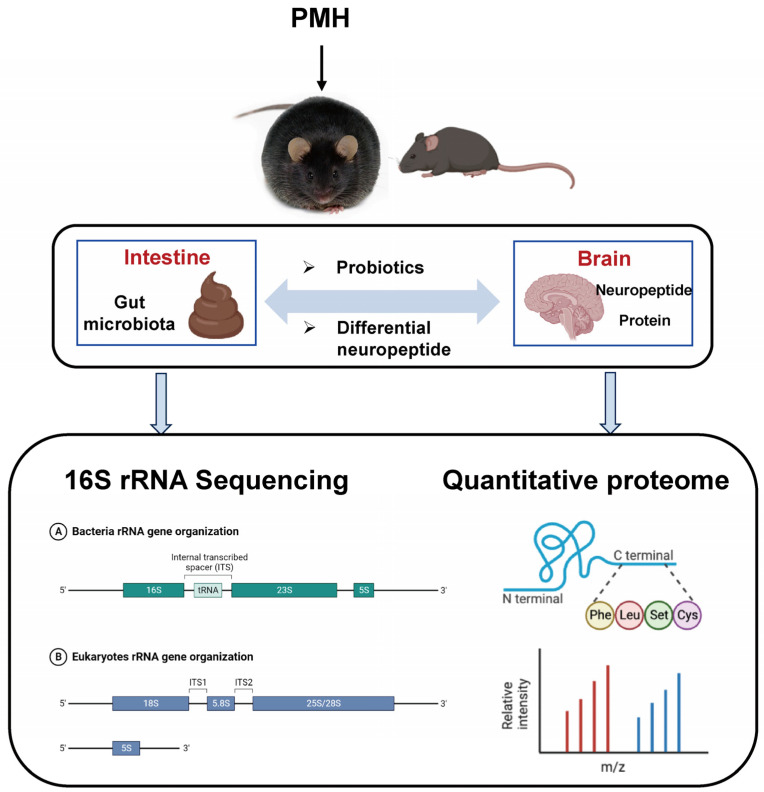
The workflow of this study.

The animal grouping and evaluation of PMH hypoglycemic activity experiments are described in previous reports [3]. The workflow is shown in Figure 5. A total of 24 male db/db mice and 6 db/m mice (7 weeks old) were purchased from GemPharmatech Co., Ltd. (Nangjing, Jiangsu, China). All experimental mice were raised in an individual ventilated cage (IVC) system under standard lighting conditions, with temperatures and humidity ranging from 21 to 24 ° C and 60% to 65%, respectively. The db/m mice were given normal feed, while the db/db group mice were given 60% high-fat feed. All groups were given free access to distilled water.

After one week of adaptation, db/db mice were randomly divided into four groups (*n* = 6): (1) DC group: high-fat diet and pure water; (2) PC group: high-fat diet and Sitagliptin Phosphate/methylin hydrochloride tablets (15/200 mg/kg per day for 4 weeks); (3) PMHL group: high-fat diet and PMH (300 mg/kg per day for 4 weeks); (4) PMHH group: high-fat diet and PMH (900 mg/kg per day for 4 weeks). The db/m mice served as normal controls (NC). Weight and food consumption were measured daily, and the entire experiment followed the regulations of the Chinese Association for Experimental Animal Science. The mice fasted for 12 h and were then euthanized at the end of the experiment.

### 3.3. Sample Collection

Feces were collected from mice in the normal control group (NC), diabetic control group (DC), and high-dose PMH group for diabetes (PMHH) at 4 weeks after administration. When collecting feces, the end of the rectum at the root of the tail was gently squeezed, and the feces were collected in a sterile EP tube after discharge, and stored at −80 °C.

Hypothalamus samples were collected from mice in the NC group, DC group, and PMHH group. The brain tissues of mice were collected and immediately frozen in liquid N2 and stored at −80 °C until they were used for analysis. 

The extraction of brain neuropeptides was slightly modified according to Chenxi Jia’s method. The tissues were manually homogenized with a tissue homogenizer. The homogenized samples were combined and centrifuged at 12,000× *g* for 20 min at 4 °C to remove the insoluble pellet. This extraction step was repeated three times. Extracts were combined and dried down in a SpeedVac vacuum concentrator to obtain a crude peptide sample.

The precipitates extracted from neuropeptides were homogenized in 4% SDS, and equal amounts of proteins were taken from each group and digested with trypsin (Promega Corporation, Madison, WI, USA) in a ratio of 100:1 at 37 °C for 18 h. The digestion was terminated by 10% TFA acidification, and the extracts were then dried down in a SpeedVac vacuum concentrator to obtain a crude protein sample. The crude peptide sample and crude protein sample were further desalinated through C18 ZipTip and resuspended in 20 μL of 0.1% formic acid aqueous solution for LC-MS/MS analysis [40].

### 3.4. DNA Extraction and Polymerase Chain Reaction Amplification

DNA extraction and polymerase chain reaction amplification were performed as shown in Supplementary Material S1.

### 3.5. 16S rRNA Sequencing and Data Analysis

The 16S rRNA sequencing and data analysis were performed as shown in Supplementary Material S2 [41].

### 3.6. Neuropeptidomic and Proteomic Analysis of Hypothalamus

The processed samples were analyzed by nano LC-MS/MS. The resulting MS/MS data were analyzed using PEAKS Studio 8.5 (BSI, Waterloo, ON, Canada) and choosing the Mollusca database (downloaded from www.uniprot.org, accessed on 23 January 2023). The database search was conducted using the SwePep neuropeptide database (www.swepep.org, accessed on 11 October 2018). 

The label-free quantitation (LFQ) method was applied to calculate the relative expression level changes by comparing the peak areas calculated using extracted ion chromatograms (XICs) using PEAKS Studio 8.5 [42]. A t-test was performed between the NC, DC, and PMHH mice groups, and any pair at *p* < 0.05 was considered statistically significant.

### 3.7. Potential Target Pathway Analysis

The Omicshare database (https://www.omicshare.com, accessed on 29 March 2023) was used to perform the GO (gene ontology) function and KEGG (Kyoto Encyclopedia of Genes and Genomes) pathway enrichment analysis on differential proteins [43].

### 3.8. Statistical Analysis

Statistical differences among the NC, DC, and PMHH treatment groups were analyzed using one-way analysis of variance (ANOVA) followed by a post hoc multiple comparisons using Fisher’s least significant difference (LSD) *t*-test. The differences in the ratios between two groups were detected by the chi-square test. A probability (*p*-value) of less than 0.05 was regarded as statistically significant.

## 4. Conclusions

In conclusion, the present study indicated that PMH ameliorated the abnormal expression of brain proteins and neuropeptides in db/db mice and regulated the composition and structure of intestinal flora in mice. Our study reveals that PMH interferes with the intestinal microbiota in mice, which affects central proteins, neuropeptides, etc., through the gut–brain axis, and intervenes in blood sugar levels through pathways such as feeding, endocrine, and hormone regulation, thereby exerting a hypoglycemic effect.

## Data Availability

The data presented in this study are available on request from the corresponding author.

## Supplementary Materials

The following supporting information can be downloaded at https://www.mdpi.com/article/10.3390/md22060249/s1: Table S1: Relative abundance of five major bacteria phyla among three groups; Table S2: All identified peptides from NC-, DC- and PMHH- brain; Table S3: Precursor proteins of brain tissue proteome differential neuropeptides; Table S4: All identified proteins from NC-, DC- and PMHH- brain; Table S5: Precursor proteins of different neuropeptide segments in mice brain tissue. S1: DNA extraction and polymerase chain reaction amplification; S2: 16S rRNA Sequencing and Data Analysis.
