# Supplementary material for: Pinctada martensii Hydrolysate Modulates the Brain Neuropeptidome and Proteome in Diabetic (db/db) Mice via the Gut–Brain Axis
Source: Mar Drugs. 2024 May 28;22(6):249. doi: 10.3390/md22060249 (PMC11204388; doi:10.3390/md22060249)
Supplement: Supplementary file 1 [file marinedrugs-22-00249-s001.zip › marinedrugs-3001000-S1 and S2.pdf]

S1:

DNA samples from mice feces were amplified by PCR and sequenced for microbial diversity. Bacterial DNA was extracted from fecal samples using the E.Z.N.A.® Soil DNA Kit (Omega Bio-Tek, United States) according to the recommendation of the manufacturer. DNA integrity and quality were evaluated by agarose gel electrophoresis (concentration of agarose gel: 1%; voltage: 5 V/cm; electrophoresis time: 20 min). And nanodrop instrument (Thermo Fisher Scientific, United States) was used to measure the concentration of DNA.

The 16S rRNA gene amplification was carried out in the lab of Majorbio Bio-pharm (Shanghai Majorbio Bio-pharm Technology Co.,Ltd). The primers were 338 F-806R (V3-V4 variable region of 16sRNA gene) with barcode. The sequence of primers was as follows: 338 F 5′-ACTCCTACGGGAGGCAGCAG-3′, 806R 5′-GGACTACHVGGGTWTCTAAT-3′. The PCR reaction system was prepared as follow: 4 μL FastPfu buffer, 2 μL dNTPs, 0.8 μL primers, 0.4 μL TransStart®FastPfu DNA polymerase (TransGen Biotech, Beijing, China), and 10 ng DNA template, supplemented with double steamed water to make the total volume be 20 μL. The conditions of PCR reaction were as follow: (1) 95 ° C 3 min; (2) a: 95 ° C 30 s; b: 55 ° C 30 s; c: 72 ° C 45 s, in a total of 27 cycles; (3) 72 ° C 10 min (Qu, Xu, Yang, Zhong, Yuan, Xian, et al., 2023). PCR products were detected by QuantiFluor™-ST blue fluorescence quantitative system (Promega Corporation, Madison, WI, USA).

S2:

Illumina platform library was constructed and sequenced by Illumina Miseq PE300 sequencing platform. Operational Taxonomic Units (OTUs) with sequence similarity over 97 % were analyzed.

Alpha diversity analysis and beta diversity analysis were calculated by QIIME and visualized by R software (V2.15.3). Alpha diversity refers to the diversity within a specific region or ecosystem. In this paper, chao is used as the metric. The Chao1 estimator reflects community richness. The Rank-Abundance curve can be used to account for two aspects of diversity, namely species richness and community

evenness. The rarefaction curve is to randomly select a certain number of sequences from the samples and count the Alpha diversity index of the corresponding samples. The extracted data volume is used as the abscess coordinate and the Alpha diversity index value is used as the ordinate to draw the curve. The adequacy of the sequencing data volume is judged according to whether the curve is flat or not. PCoA was applied to examine dissimilarities in community composition and microbiota abundance was constructed based on the unweighted UniFrac distance metric. All diversity measurements were conducted on OTU tables. Furthermore, LEfSe was performed by non-parametric factorial KW sum-rank test in order to identify potential microbial biomarkers associated with particular interventions. The Student's test was used to test the difference between the index groups. The community composition of each group was analyzed based on phylum level.
